# Membrane-Active Antibacterial Agents Based on Calix[4]arene Derivatives: Synthesis and Biological Evaluation

**DOI:** 10.3389/fchem.2022.816741

**Published:** 2022-02-08

**Authors:** Shanfang Fang, Yuan-Ye Dang, Haizhou Li, Hongxia Li, Jiayong Liu, Rongcui Zhong, Yongzhi Chen, Shouping Liu, Shuimu Lin

**Affiliations:** The Fifth Affiliated Hospital and Key Laboratory of Molecular Target and Clinical Pharmacology and the State Key Laboratory of Respiratory Disease, School of Pharmaceutical Sciences, Guangzhou Medical University, Guangzhou, China

**Keywords:** antimicrobial agents, Calix[4]arene derivatives, membrane-active, peptidomimetics, bacterial resistance, gram-positive bacteria

## Abstract

Bacteria have developed increasing resistance to currently used antimicrobial agents. New classes of antimicrobial drugs are urgently required to fight drug-resistant pathogens. Here, we designed and synthesized a series of calix[4]arene derivatives as antibacterial agents by biomimicking the structural properties and biological functions of antibacterial peptides. After introducing cationic hydrophilic moieties and preliminary structural optimization, we obtained a lead compound (**16**) that exhibited excellent antibacterial activity against Gram-positive bacteria, low toxicity toward mammalian cells and poor hemolytic activity. The antibacterial mechanism studies showed that compound **16** can destroy bacterial cell membrane directly, leading to bacterial death and a low tendency to develop bacterial resistance.

**Graphical Abstract:**
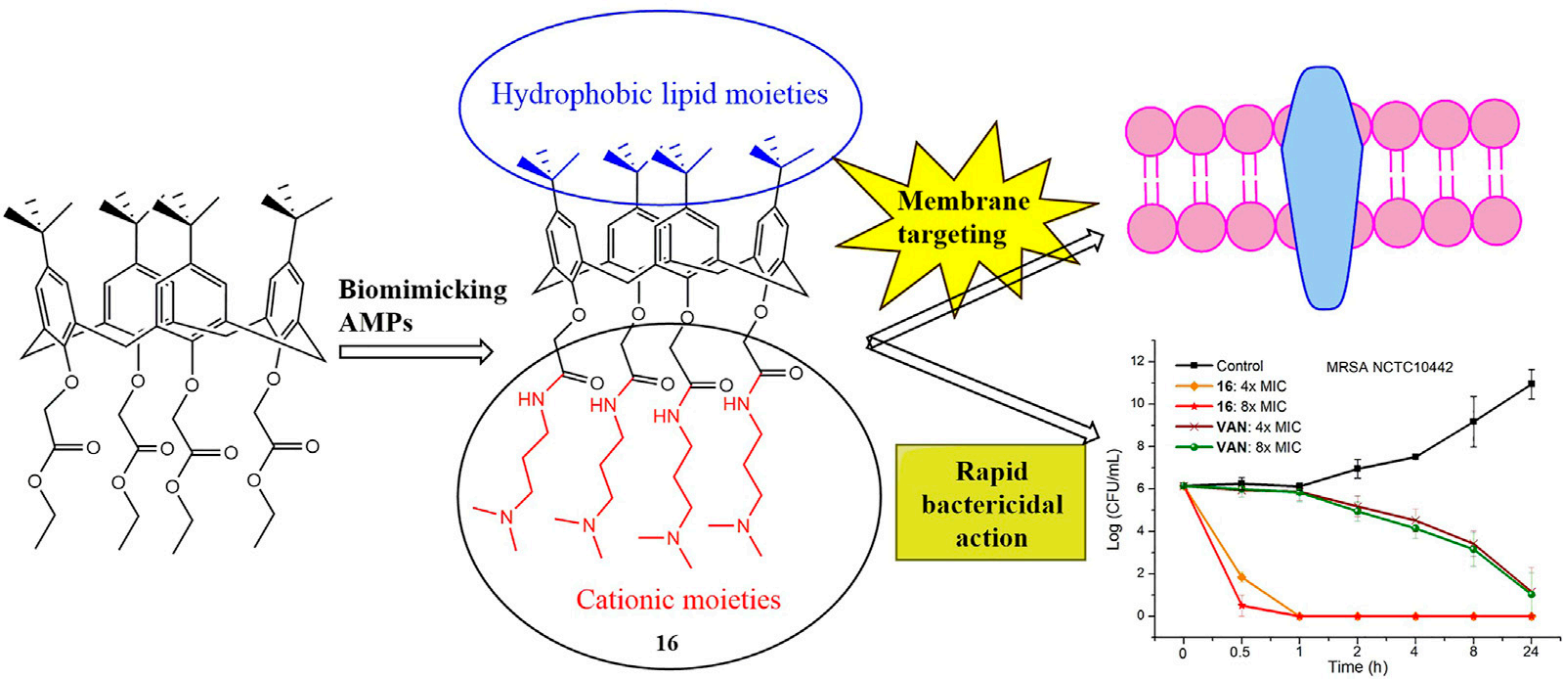


## Introduction

The development of multi-drug resistant bacteria and the decrease in the discovery of new antibiotics pose a major threat to public health in the 21st century (Lyddiard et al., 2016; Woolhouse et al., 2016; Monserrat-Martinez et al., 2019). In addition, due to the rapid development of antimicrobial resistance, short medication cycle, and low profits of antimicrobial agents, many pharmaceutical companies have greatly reduced their investment in the development of antimicrobial agents, which could slow the discovery of new antibiotics (Livermore, 2011; Rogers et al., 2012). The ESKAPE pathogens, including E*nterococcus faecium*, *Staphylococcus aureus*, *Klebsiella pneumoniae*, *Acinetobacter baumannii*, *Pseudomonas aeruginosa*, and *Enterobacter* spp.*,* are spreading and becoming more resistant to many commonly used antibacterial agents (Rice, 2008). Currently, most newly approved antibiotics are likely to develop rapid resistance as most of them belong to known classes of antibiotics, based on the molecular scaffolds of traditional antibiotics (Molchanova et al., 2017). In 2017, the World Health Organization (WHO) released a priority list of drug-resistant pathogens to guide the development of new antibiotics and warned that there was a serious lack of new antibiotics to treat the growing number of drug-resistant infections (Kmietowicz, 2017). Antimicrobial resistance causes increases in mortality, morbidity, length of hospitalization and cost of healthcare (Maragakis et al., 2008). If effective measures are not taken to combat antibiotic resistance, we will return to the “pre-antibiotic era,” and most surgical procedures will not be safe to implement.

Antimicrobial peptides (AMPs), also known as host defense peptides, exist in various life forms from microorganisms to humans. Most of them are positively charged and possess broad-spectrum antimicrobial activity against bacteria, viruses and fungi (Koh et al., 2015; Lázár et al., 2018; Arbour et al., 2020; Thapa et al., 2020). Compared with conventional antibiotics, AMPs have several obvious advantages, such as rapid bactericidal action (Li et al., 2017), immunomodulatory activity (Jenssen et al., 2006), synergistic effects with antibacterial agents (Zasloff, 2002), and low probability of developing bacterial resistance (Wimley and Hristova, 2011). However, the progress in the clinical application of AMPs has been hindered by the sensitivity to proteolytic enzymes, poor *in vivo* efficacy and poor pharmacokinetic properties (Adessi and Soto, 2002; Marr et al., 2006; Costa et al., 2019). Small peptidomimetics that mimic the chemical structure and biological function of AMPs can avoid most of the defects of AMPs (Hickey et al., 2015; Nizalapur et al., 2017). Several small peptidomimetics have shown great application potential, such as Brilacitine and LTX-109 that have successfully entered or completed Phase II clinical trials (Isaksson et al., 2011; Mensa et al., 2014; Kowalski et al., 2016; Kuppusamy et al., 2019).

Calixarene derivatives have been found to contain various biological activities, such as antiviral, antibacterial, antifungal and anticancer activities (Yousaf et al., 2015; Nasuhi Pur, 2016). In addition, they do not display obvious cytotoxicity and immunogenicity (Ma and Zhao, 2015; Pur, 2021). Due to their versatility, low cost and unique three-dimensional structures, calixarene could serve as an ideal molecular scaffold for the design and optimization of drug molecules (Hanna et al., 2003; Mokhtari and Pourabdollah, 2013). Calix[4]arene derivatives have attracted special attention owing to the cheap and easy availability, unique structural characteristics and easy modification (Naseer et al., 2017; Bono et al., 2018). The functionalized basket cavity of calix[4]arene is suitable for small ions and neutral molecules, and calix[4]arene can provide excellent conditions for the incorporation of other moieties via the hydroxyl groups (Naseer et al., 2017). The antitumor agent calix[4]arene compound OTX008 that targets human galectin-1 is being evaluated in phase I clinical trial, indicating that calixarene has great potential as a molecular skeleton of drugs (Läppchen et al., 2015). However, to our knowledge, there are no other calixarene-based drugs have entered clinical trials at present (Nimse and Kim 2013). As reported, calixarene derivatives have great potential as antibacterial agents and could avoid cross-resistance with existing antibacterial agents due to their different molecular structures from existing antibacterial agents (Shurpik et al., 2020).

In this work, by biomimicking AMPs, we designed and synthesized a series of calix[4]arene derivatives as membrane-active antibacterial agents. The most promising compound **16** showed potent antibacterial activity against Gram-positive bacteria, including methicillin-resistant *Staphylococcus aureus* (MRSA)*.* No bacterial resistance was observed for compound **16** in the laboratory simulation of the drug resistance study. The commercial reagents 4-tert-butylcalix[4]arene, and tetraethyl 4-tert-butylcalix[4]arene-O,O′,O″,O‴-tetraacetate were used as the starting materials, and then cationic moieties were introduced to the calix[4]arene scaffold to form cationic amphiphilic structures. The introduced cationic moieties are beneficial to identify bacterial cells, which can promote the interaction between cationic calix[4]arene compounds and negatively charged bacterial membranes via electrostatic action, while the hydrophobic tert-butyl groups can facilitate the insertion of calix[4]arene compounds into bacterial phospholipid bilayer membranes, leading to the change in the permeability of bacterial cell membranes and the death of bacteria. The difference in cell membrane composition between eukaryotes and bacteria is very conducive to improving the selectivity of cationic amphiphilic calix[4]arene derivatives. Bacterial membranes are rich in anionic lipids (Malmsten, 2014), while the membrane surfaces of eukaryotes mainly contain zwitterionic phospholipids including phosphatidylethanolamine, phosphatidylcholine, and sphingomyelin (Pasupuleti et al., 2012). After a series of structural modifications, the cationic hydrophilic moieties of calix[4]arene derivatives were fine-tuned to obtain potent antibacterial agents with good antibacterial activity and low toxicity. Finally, the time-kill kinetics, drug resistance development, *in vitro* cytotoxicity toward mammalian cells, and the antibacterial mechanism were studied. These findings suggest that this design strategy for calix[4]arene-based AMPs mimics is very conducive to developing new antibacterial agents to fight drug-resistant bacteria.

## Results and Discussion

### Design and Synthesis of Calix[4]arene-Based Antibacterial Derivatives

**SCHEME 1:**
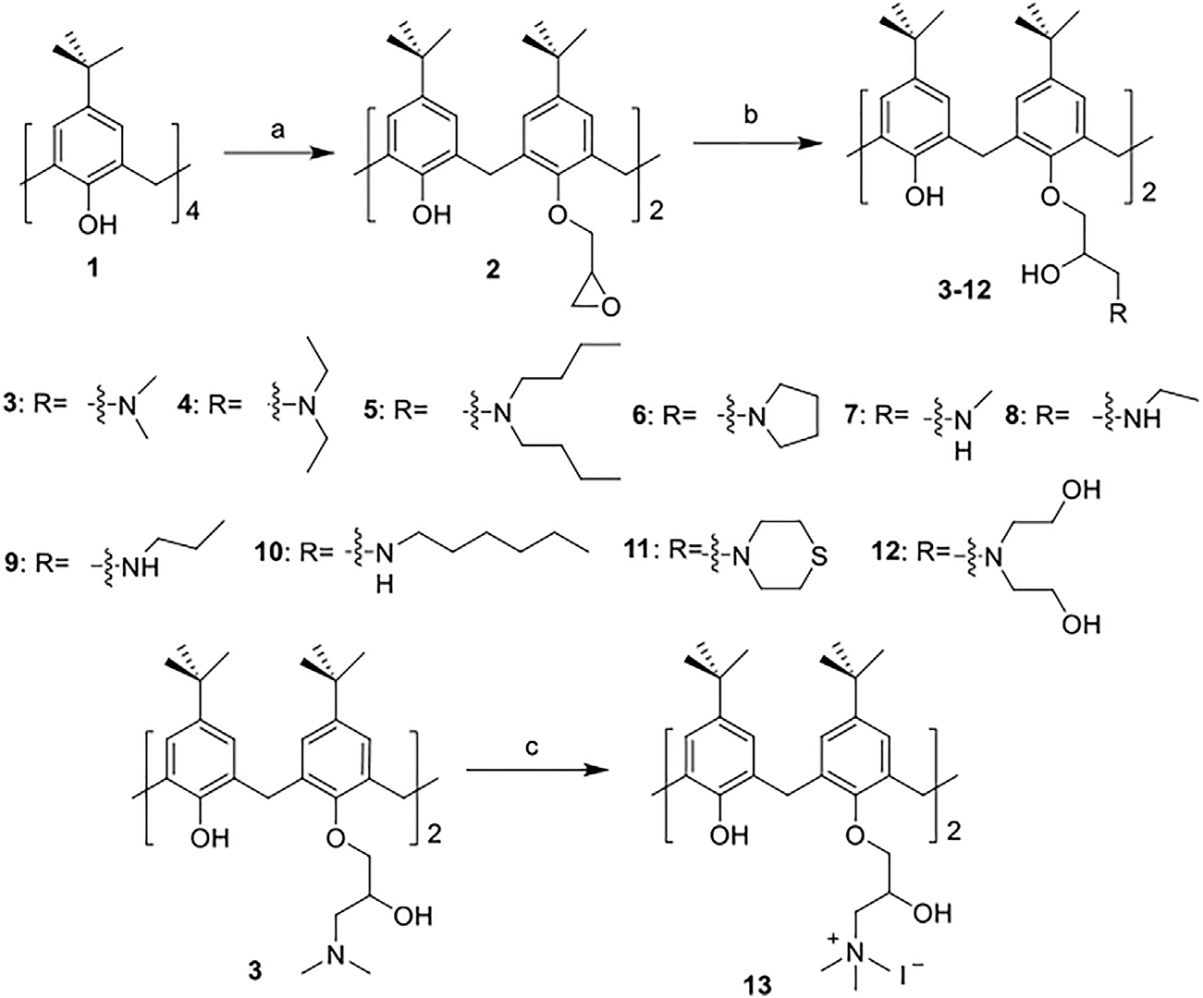
Synthesis of cationic amphiphilic calix[4]arene derivatives **2–13**. Reagents and conditions: (a) epibromohydrin, KI, K_2_CO_3_, acetonitrile, 85°C, reflux, 10–12 h (b) corresponding amines, CH_3_OH, 65°C, reflux, 3 h. (c) Iodomethane, CH_3_OH, room temperature (RT), overnight.

**SCHEME 2:**
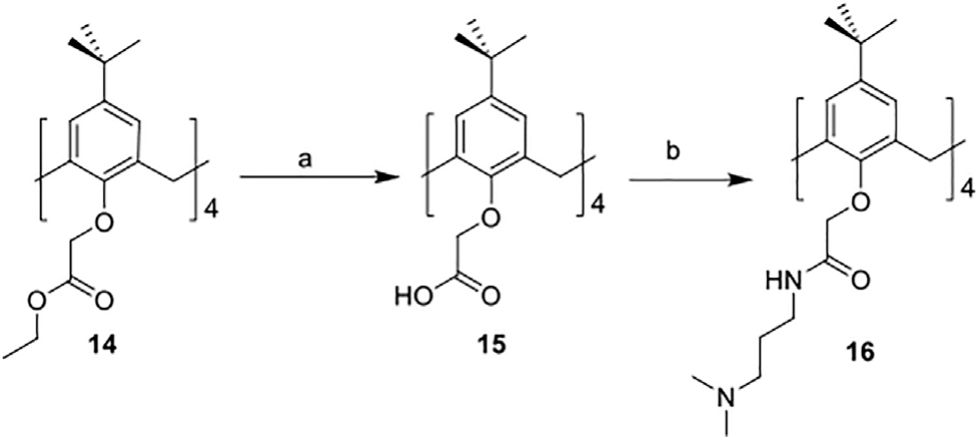
Synthesis of cationic amphiphilic calix[4]arene derivatives **15–16.** Reagents and conditions: (a) LiOH, THF/water, RT, 1.5 h (b) 3-dimethylaminopropylamine, DMTMM, DIPEA, DMF, RT, 12 h.

**SCHEME 3:**
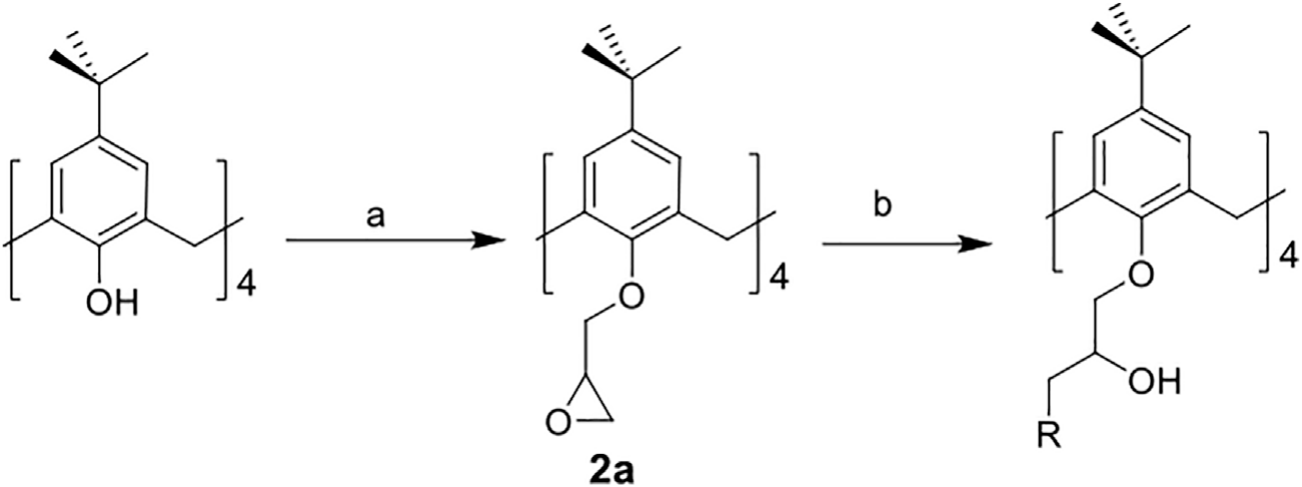
Synthesis of calix[4]arene derivative **2a.** Reagents and conditions: (a) epibromohydrin, KI, K_2_CO_3_ or potassium tert-butoxide, acetonitrile, 85°C, reflux. (b) corresponding amines, CH_3_OH, 65°C, reflux.

The synthetic routes for synthesizing various amphiphilic calix[4]arene analogs are shown in Schemes 1, 2. The starting material 4-tert-butylcalix[4]arene was treated with epibromohydrine together with K_2_CO_3_ to yield intermediate compound **2**. Compounds **3–12** were then obtained by the treatment of compound **2** with corresponding amines. Polyamine compounds often become polycations at physiological pH and then exert their biological activity. The Nitrogen atoms containing lone pairs of electrons in polyamines are easily protonated to be positively charged. Compound **3** was coupled with iodomethane to afford compound **13**. The starting materials tetraethyl 4-tert-butylcalix[4]arene-O,O′,O″,O‴-tetraacetate was hydrolyzed by LiOH to produce compound **15**. Then the acid **15** was reacted with 3-dimethylaminopropylamine in the presence of 4-(4,6-dimethoxy-1,3,5-triazin-2-yl)-4-methylmorpholinium chloride (DMTMM) to provide compound **16**. In fact, we initially planned to design and synthesize a four-arm modified intermediate epoxy compound **2a** (Scheme 3), and then **2a** reacted with the corresponding amines to prepare a series of amphiphilic cationic four-arm modified calixarene derivatives as antibacterial compounds (Scheme 3). We tried many different conditions, but failed to synthesize **2a**. All synthesized compounds were characterized by H NMR, C NMR, and HRMS.

### *In Vitro* Antibacterial and Hemolytic Activity

The *in vitro* antibacterial activity of the calix[4]arene derivatives was evaluated against three Gram-positive bacteria, including *S. aureus* ATCC29213, MRSA NCTC10442 and MRSA N315. The *in vitro* antibacterial activity was assessed by minimum inhibitory concentrations (MICs). The HC_50_ value (concentration of compounds required to lyse 50% of rabbit red blood cells) was used to evaluate the hemolytic activity of the synthesized compounds toward rabbit red blood cells (Table 1).

**TABLE 1:** *In vitro* anti-Gram-positive bacterial activities and hemolytic activities of calix[4]arene derivatives **1–16.**

Compound	MIC, µg/ml (µM)			HC_50_, µg/ml (µM)
	*S. aureus* ATCC29213	MRSA N315	MRSA NCTC10442	
**1**	>50 (>77)	>50 (>77)	>50 (>77)	>200 (>308)
**2**	>50 (>66)	>50 (>66)	>50 (>66)	>200 (>263)
**3**	13 (15)	13 (15)	13 (15)	>200 (>235)
**4**	25 (28)	13 (14)	25 (28)	>200 (>220)
**5**	>50 (>49)	>50 (>49)	>50 (>49)	>200 (>196)
**6**	50 (55)	50 (55)	>50 (>55)	>200 (>221)
**7**	4.7 ± 2 (5.7 ± 2)	1.6 (1.9)	3.1 (3.8)	151 ± 5 (183 ± 6)
**8**	1.6 (1.8)	1.2 ± 0.4 (1.4 ± 0.5)	1.6 (1.8)	123 ± 8 (144 ± 9)
**9**	2.3 ± 0.8 (2.6 ± 0.9)	2.3 ± 0.8 (2.6 ± 0.9)	4.7 ± 2 (5.3 ± 2)	191 ± 2 (217 ± 3)
**10**	>50 (>52)	>50 (>52)	>50 (>52)	>200 (>208)
**11**	>50 (>52)	>50 (>52)	>50 (>52)	>200 (>207)
**12**	>50 (>52)	>50 (>52)	>50 (>52)	>200 (>206)
**13**	3.1 (2.8)	3.1 (2.8)	3.1 (2.8)	16 ± 1 (14 ± 1)
**14**	>50 (>50)	>50 (>50)	>50 (>50)	>200 (>201)
**16**	1.6 (1.3)	1.6 (1.3)	3.1 (2.6)	>200 (>164)
**vancomycin**	0.78 (0.50)	1.6 (1.1)	1.6 (1.1)	ND^a^

a Not determined.

To investigate the effects of different secondary amine substituents on the antibacterial and hemolytic activities of the calix[4]arene derivatives, compounds **7–10** were synthesized. Compounds **7–9** displayed good antibacterial activities against Gram-positive bacterial strains tested (MICs = 1.2–4.7 μg/ml) and weak hemolytic activity, with HC_50_ values in the range of 123–191 μg/ml. However, n-hexylamine-coupled compound **10**, a more hydrophobic compound, showed very poor antibacterial activity (MICs > 50 μg/ml) and very poor hemolytic activities (HC_50_ > 200 μg/ml). The anti-Gram-positive bacterial activities of these compounds are determined by the hydrophobic side chains connected to positive charge centers. These results suggested that the secondary amine substituents with short hydrophobic side chains can greatly improve the antibacterial activity of calix[4]arene derivatives, and also slightly increase the hemolytic activity of calix[4]arene derivatives.

Next, compounds **3–6**, **11–12**, and **16** were used to explore the effects of different types of tertiary amine substitutions on the hemolytic and antibacterial activity of amphiphilic calix[4]arene derivatives. Dimethylamine-coupled compound **3** and diethylamine-coupled compound **4** showed moderate antibacterial activity (MICs = 13–25 μg/ml). However, dibutylamine-coupled compound **5** did not display any antibacterial activities even at the highest tested concentration of 50 μg/ml. This result indicated that tertiary amine substituted calix[4]arene derivatives with long alkyl side chains would lead to a significant decrease or even loss in biological activity. When introducing pyrrolidine (**6**), very weak antibacterial activity was observed (MICs ≥ 50 μg/ml). 3-Dimethylaminopropylamine-containing compound **16** with four-arm functionalization displayed enhanced antibacterial activity, with MICs in the range of 1.6–3.1 μg/ml. Compound **16** was also used to investigate the effect of four-arm modification and two-arm modification on the biological activity of amphiphilic cationic calixarene derivatives. We found that the membrane selectivity of four-arm modified compound **16** was higher than that of two-arm modified calixarene derivatives. Owing to the low pKa value of the introduced cationic group, compound **11** containing thiomorpholine (pKa of free thiomorpholine = 9.0) (Jones et al., 2017), and compound **12** containing diethanolamine (pKa of free diethanolamine = 8.9) (Singh et al., 2011), exhibited very poor antibacterial activity against Gram-positive bacteria (MICs > 50 μg/ml). It manifested that the amine substituents with low pKa value were harmful to the antibacterial activity of calix[4]arene derivatives. No hemolytic activity (HC_50_ > 200 μg/ml) was observed for these eight compounds, suggesting that tertiary amine substitutions had little effect on the hemolytic activity of calix[4]arene derivatives. Tertiary amine **3** was reacted with iodomethane to yield quaternary ammonium salt **13** that was used to investigate the effect of quaternary ammonium salt substitution on the antibacterial and hemolytic activities of amphiphilic calix[4]arene derivatives. Compared with the precursor compound **3**, both the activity against Gram-positive bacteria and the hemolytic activity of compound **13** increased obviously, with MICs of 3.1 μg/ml and HC_50_ of 16 ± 1 μg/ml. These results suggested that quaternary ammonium salt substitutions had a significant effect on the antibacterial activity and hemolytic activity of calix[4]arene derivatives.

From the preliminary structure-activity relationship (SAR) study of amphiphilic calix[4]arene derivatives, we found that several structural parameters have significant effects on the antibacterial and hemolytic activities, including the types of amine substituents, the length of the carbon chain connected to the positive charge center and the pKa value of the cationic group. The introduction of amine groups with long hydrophobic side chains would lead to the loss in antibacterial activity of calix[4]arene compounds (MIC > 50 μg/ml). In addition, poor antibacterial activity was observed when low pKa amine groups were incorporated. 3-Dimethylaminopropylamine coupled compound **16** displayed excellent anti-Gram-positive bacterial activity (MICs = 1.6–3.1 μg/ml) and very weak hemolytic activity (HC_50_ > 200 μg/ml). The cationic moieties play an important role in the interaction with negatively charged bacterial membranes. The incorporation of cationic groups enables the cationic calix[4]arene derivatives to act on negatively charged bacterial cell membranes through electrostatic interaction, which is helpful for calix[4]arene derivatives to distinguish bacterial cell membranes from mammalian cell membranes. Among all the synthesized compounds, only compound **16** with four-arm tertiary amines functionalized showed excellent antimicrobial activity and very poor hemolytic activity.

### Time-Kill Kinetics

**FIGURE 1:**
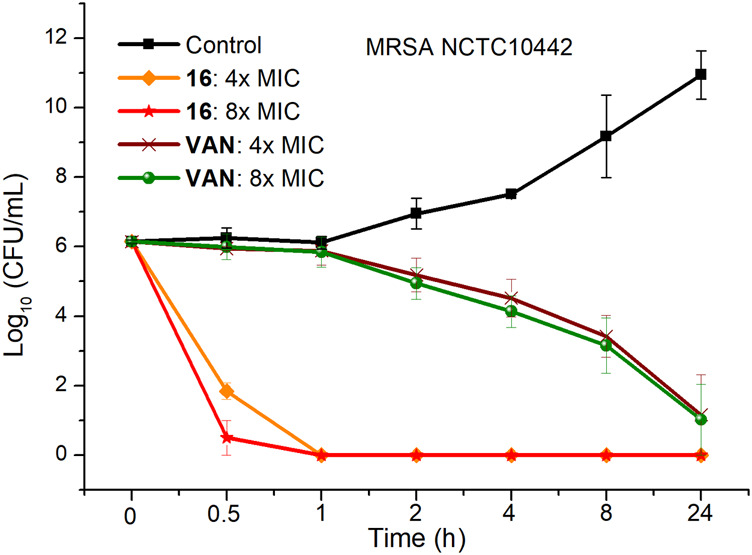
Time-kill kinetics of compound **16** and vancomycin against MRSA NCTC10442.

To investigate the bactericidal performance of calix[4]arene derivatives, we determined the time-kill kinetics curves of compound **16** against MRSA NCTC10442 at different concentrations (4× and 8× MIC). As shown in Figure 1, compound **16** reduced 4.3 and 5.6 log bacteria (killing > 99.99% of MRSA NCTC10442) within 0.5 h at 4× and 8× MIC, respectively, indicating that compound **16** exhibited rapid bactericidal activity. In contrast, the commercial vancomycin only achieved 1.6 and 2.0 log bacterial reductions within 4 h at 4× and 8× MIC, respectively. These findings indicated that compound **16** showed rapid bactericidal action that will help to reduce the treatment time of bacterial infection and the probability of developing bacterial resistance.

### Resistance Development Study

**FIGURE 2:**
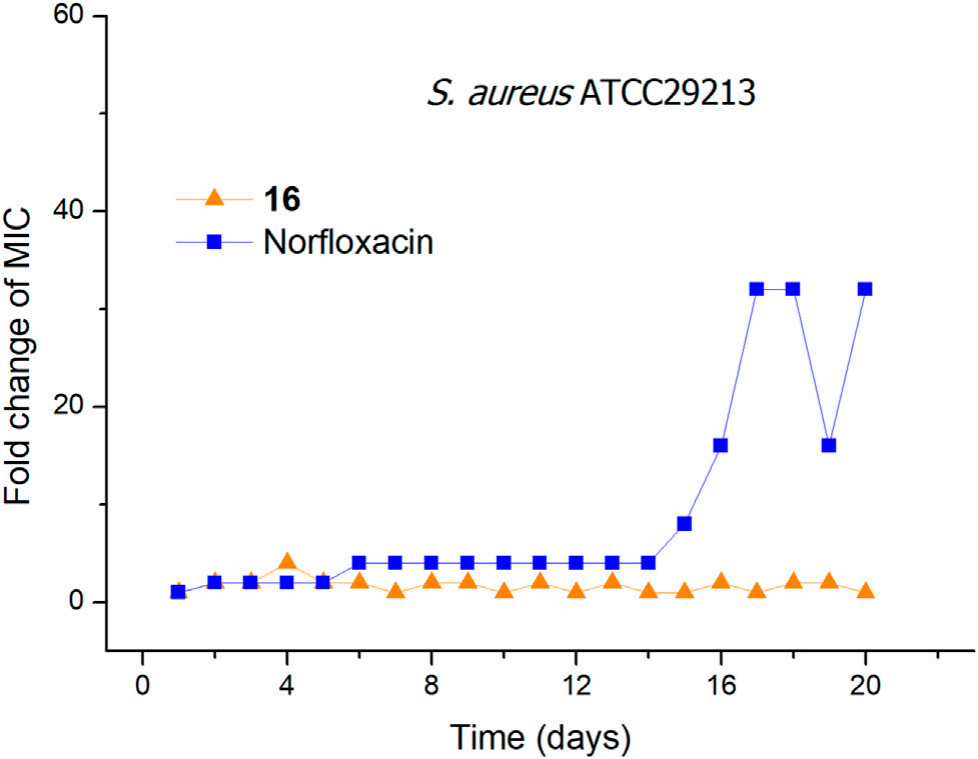
Bacterial resistance studies of compound **16** and norfloxacin against *S. aureus* ATCC29213.

The ability to overcome bacterial resistance has become an important criterion to evaluate a new antibacterial drug (Gajdács, 2019). The rapid development of bacterial resistance to antimicrobials is a major threat to public health in the 21st century (Walsh, 2000; O’Neill, 2008; Piddock, 2012). The membrane-targeting antibacterial agents can effectively avoid or slow down the development of bacterial resistance. To evaluate the tendency of bacterial resistance of compound **16**, a laboratory simulation study on the drug resistance of compound **16** was carried out. In the presence of a sublethal concentration of compound **16** or norfloxacin, *S. aureus* ATCC29213 was consecutively passaged for 19 days. As shown in Figure 2, after 19 passages, no more than a 4-fold increase in the MIC values was observed for compound **16**. In contrast, the MIC value of norfloxacin was increased by 32-fold after 17 passages, indicating that norfloxacin could rapidly induce bacterial resistance. The reason may be that the bacterial membrane is a conservative component in the evolution of bacterial cells and determines the phenotype, it is difficult for bacteria to keep alive with great changes in the composition of the cell membranes (Zasloff, 2002; Yeaman and Yount, 2003). These results demonstrated that compound **16** has obvious advantages over conventional antibacterial drugs, and can avoid the occurrence of developing bacterial resistance.

### Antibacterial Mechanism Studies

**FIGURE 3:**
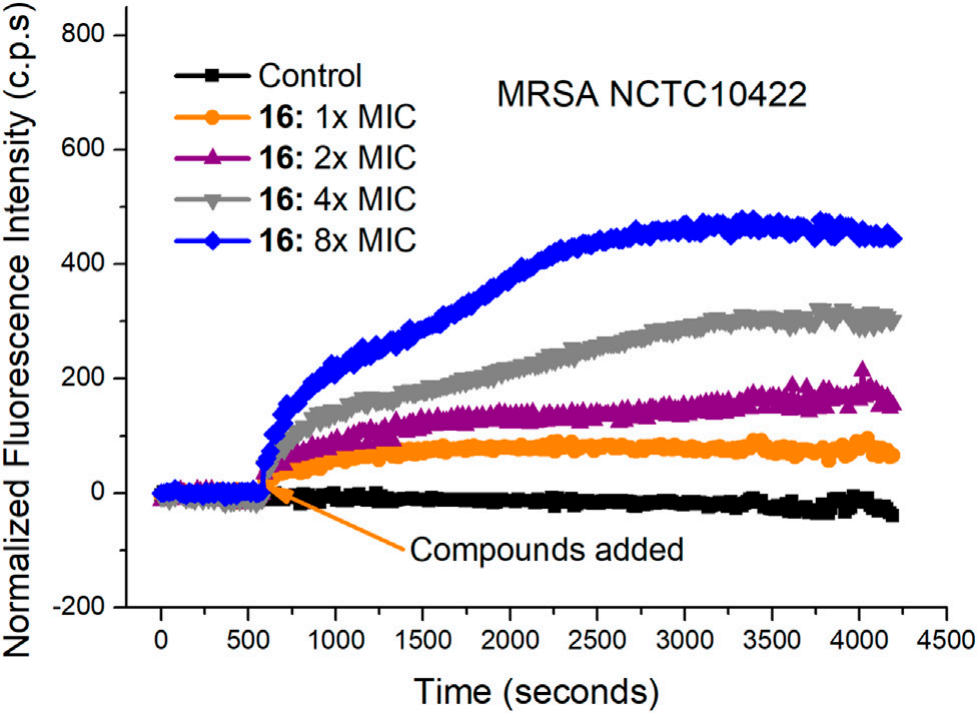
Cytoplasmic membrane permeabilization of compounds **16** against MRSA NCTC10442.

The rapid bactericidal action of compound **16** may be based on the membrane-active mode of action. To verify this hypothesis, SYTOX Green dye was used to study the effect of compound **16** on the bacterial cell membrane. SYTOX Green is a green nucleic acid dye that can easily penetrate the damaged bacterial cell membrane but cannot penetrate the living cell membrane with a complete membrane structure (Roth et al., 1997; Thakur et al., 2015). After binding with intracellular nucleic acid, its fluorescence intensity will be significantly enhanced. As shown in Figure 3, when MRSA NCTC10442 were treated with compound **16** at four different concentrations (1×, 2×, 4× and 8× MIC), the fluorescence intensity increased notably. The results indicated that compound **16** could increase the permeability of Gram-positive bacterial cell membrane, and then destroy the integrity of bacterial cell membranes, causing the leakage of the cellular contents and bacterial cell death.

**FIGURE 4:**
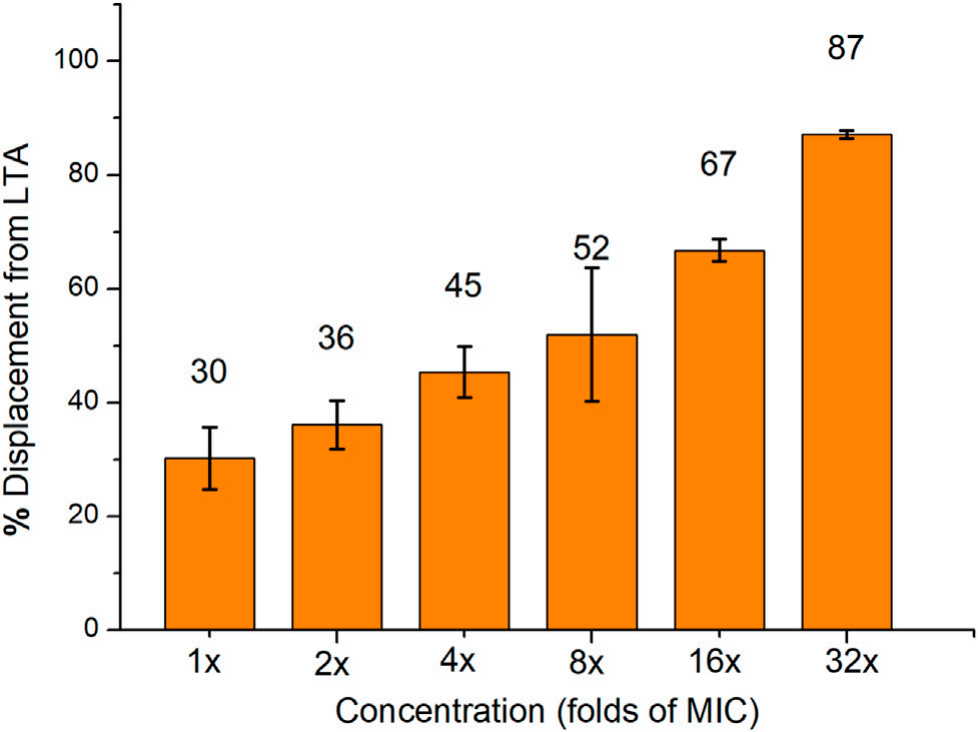
The displacement of BODIPY-TR-cadaverine from LTA was caused by compound **16**. Data are presented as the average value of two independent experiments.

The interaction between compound **16** and lipoteichoic acid (LTA) which was one of the main components of Gram-positive bacteria was also explored. The binding affinity between compound **16** and LTA was determined using the BODIPY-TR-cadaverine displacement assay. The fluorescence intensity of the probe was quenched when it was bound to LTA (Swain et al., 2019). When the probe is replaced by compound **16** and dissolved in solution, the fluorescence will be significantly enhanced. As shown in Figure 4, a proper amount of BODIPY-TR-cadaverine (30 ± 5%) was replaced by 1× MIC compound **16** from LTA. And compound **16** can replace a considerable amount of BODIPY-TR-cadaverine from LTA at 16× MIC (67 ± 2%), indicating that compound **16** could interact with LTA on the bacterial surface *via* a concentration-dependent manner.

### *In Vitro* Cytotoxicity Evaluation

**FIGURE 5:**
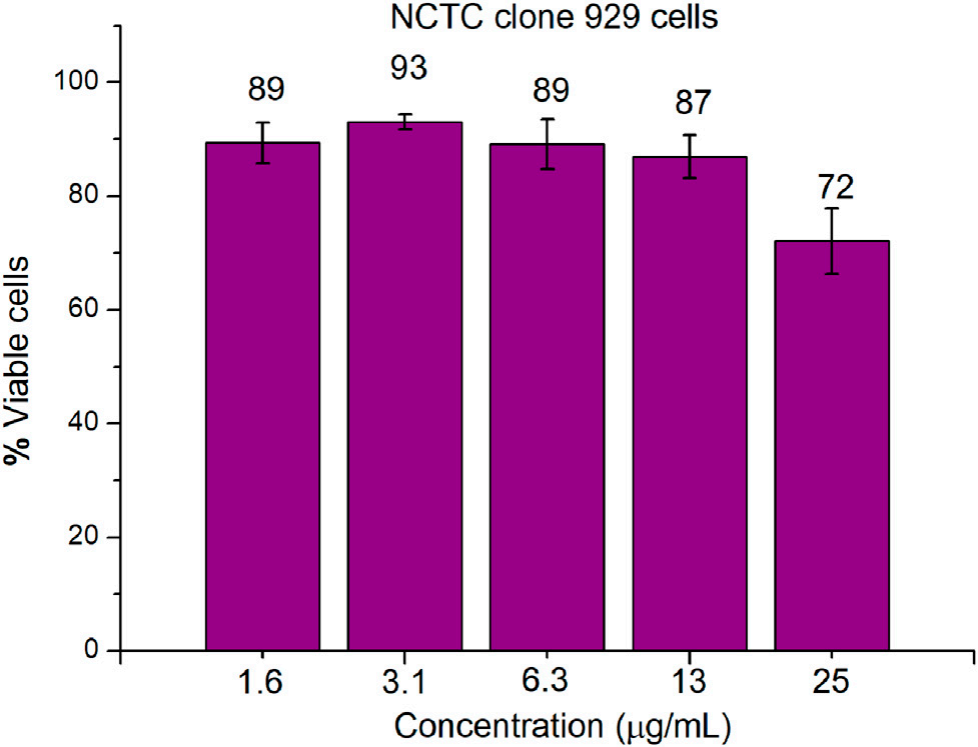
Cytotoxicity of compound **16** against mouse NCTC clone 929 cells using CCK-8 assays.

An important parameter in the development of antimicrobial drugs is their ability to selectively act on microbial cells rather than mammalian cells. CCK-8 assay was used to evaluate the *in vitro* cytotoxicity of compound **16** toward mammalian cells (mouse fibroblast NCTC clone 929). As shown in Figure 5, compound **16** showed very low cytotoxicity toward mouse fibroblast NCTC clone 929 cells at the concentration of ≤ 25 μg/ml (8–16× MIC). 72 ± 6% viability of mouse fibroblasts was observed for compound **16** at 25 μg/ml. In general, when considering the low MIC values of compound **16** against Gram-positive bacterial strains, these cytotoxicity results are very encouraging.

## Conclusion

In summary, we have designed and synthesized a series of calix[4]arene-based antibacterial agents, and have evaluated the *in vitro* antibacterial and hemolytic activity of these synthesized compounds. The incorporation of different cationic groups can effectively improve the antibacterial activity of calix[4]arene derivatives. The most promising compound **16** showed excellent activity against all tested Gram-positive bacterial strains. The antibacterial mechanism studies have shown that compound **16** could destroy the integrity of Gram-positive bacteria, causing the leakage of cell contents and leading to bacterial cell deaths. Membrane active mode of action and rapid bactericidal ability can effectively reduce the probability of bacterial drug resistance. In general, we have synthesized a class of calix[4]arene derivatives as membrane-active antibacterial agents, which provided a new design idea for the development of new membrane-active antibacterial agents.

## Materials and Methods

### Chemistry

All chemicals and solvents were purchased from commercial suppliers and used directly without further purification. 4-Tert-butylcalix[4]arene, and tetraethyl 4-tert-butylcalix[4]arene-O,O′,O″,O‴-tetraacetate were purchased from Aladdin Biochemical Technology Co., Ltd. The completion of all reactions was monitored by thin-layer chromatography (Merck silica gel 60 F254). ^1^H and ^13^C NMR spectra were recorded on the JEOL 400 MHz spectrometer operating at frequencies of 400 and 100 MHz, respectively. Chemical shifts (δ) are given in ppm, and coupling constants (J) are given in Hz. HRMS spectra were recorded on a Thermo DFS mass spectrometer. The final products were purified by preparative high-performance liquid chromatography on an Agilent 1260 Integrated system (C18 column, YMC-Pack, 20 mm × 150 mm, 5 µm), using methanol and distilled water (both containing 0.1% formic acid) as the gradient elution.

#### 1^5^,3^5^,5^5^,7^5^-Tetra-tert-butyl-3^2^,7^2^-bis(oxiran-2-ylmethoxy)-1,3,5,7(1,3)-tetrabenzenacyclooctaphane-1^2^,5^2^-diol (2)

4-Tert-butylcalix[4]arene (200 mg, 0.31 mmol) was dissolved in anhydrous acetonitrile by ultrasonic, and then epibromohydrin (202 μl, 2.5 mmol), potassium iodide (20 mg, 0.12 mmol) and potassium carbonate (170 mg, 1.2 mmol) were added. The reaction mixture was refluxed at 85°C overnight. After completion of the reaction, the mixture was diluted with ethyl acetate and washed twice with water. The organic phase was concentrated under reduced pressure, and the crude product was purified by silica gel chromatography (petroleum ether: ethyl acetate, 4:1, v:v) to give compound **2** as a white solid (141 mg, 70%). ^1^H NMR (400 MHz, CDCl_3_) δ 7.18–7.09 (m, 2H), 7.08–7.03 (m, 4H), 6.81 (s, 4H), 4.37–4.21 (m, 6H), 4.10–4.03 (m, 2H), 3.55 (s, 2H), 3.37–3.28 (m, 4H), 3.11–2.89 (m, 4H), 1.30–1.26 (m, 18H), 1.02–0.92 (m, 18H). ^13^C NMR (100 MHz, CDCl_3_) δ 150.55, 150.50, 149.56, 149.52, 147.25 (2×C), 141.64, 141.61, 141.58, 132.78, 132.66, 132.62, 132.48, 127.93, 127.79, 127.73, 127.64, 125.80, 125.71, 125.59, 125.22, 125.19, 125.15, 125.10, 76.04, 75.75, 60.47, 50.28, 44.77, 44.73, 34.02, 34.01, 33.89 (2×C), 31.75 (6×CH_3_), 31.62 (2×CH_2_), 31.59 (2×CH_2_), 31.07 (6×CH_3_). HRMS (ESI+): calculated for C_50_H_65_O_6_ [M + H]^+^ 761.4781, found 761.4762.

#### 1^5^,3^5^,5^5^,7^5^-Tetra-tert-butyl-3^2^,7^2^-bis(3-(dimethylamino)-2-hydroxypropoxy)-1,3,5,7(1,3)-tetrabenzenacyclooctaphane-1^2^,5^2^-diol (3)

To a solution of **2** (38 mg, 0.050 mmol) in methanol (5.0 ml), dimethylamine (1.0 ml) was added, and the reaction mixture was refluxed at 65°C for 3 h. After completion of the reaction, the reaction mixture was directly distilled under reduced pressure. The crude product was purified by HPLC to give compound **3** as a white solid (22 mg, 59%). ^1^H NMR (400 MHz, CD_3_OD) δ 7.19–7.11 (m, 8H), 4.61–4.37 (m, 2H), 4.35–4.24 (m, 4H), 4.23–4.08 (m, 3H), 4.06–3.92 (m, 2H), 3.65–3.36 (m, 6H), 3.22–3.13 (m, 1H), 2.82 (s, 12H), 1.23 (s, 18H), 1.14–1.10 (m, 18H). ^13^C NMR (100 MHz, CDCl_3_) δ 149.93, 149.88, 149.04, 148.89, 147.85, 147.71, 142.57, 142.51, 132.80, 132.64, 132.56, 132.47, 127.84, 127.81, 127.79, 127.43, 126.24, 125.96, 125.82, 125.62, 125.49, 125.45, 125.36, 100.00, 78.36, 78.29, 66.13, 65.73, 61.26, 61.17, 44.62 (2×CH_3_), 44.55 (2×CH_3_), 34.16, 34.10, 33.95 (2×C), 31.72 (2×CH_2_), 31.71 (2×CH_2_), 31.10 (6×CH_3_), 31.07 (6×CH_3_). HRMS (ESI+): calculated for C_54_H_79_N_2_O_6_ [M + H]^+^ 851.5938, found 851.5923.

#### 1^5^,3^5^,5^5^,7^5^-Tetra-tert-butyl-3^2^,7^2^-bis(3-(diethylamino)-2-hydroxypropoxy)-1,3,5,7(1,3)-tetrabenzenacyclooctaphane-1^2^,5^2^-diol (4)

Compound **4** was prepared from **2** (37 mg, 0.049 mmol) and diethylamine (1.0 ml), following the procedure used to prepare **3.** The product was obtained as a white solid (28 mg, 75%). ^1^H NMR (400 MHz, CDCl_3_) δ 8.20 (s, 1H), 7.82–7.72 (m, 1H), 7.10–6.99 (m, 4H), 6.98–6.89 (m, 2H), 6.88 (s, 2H), 4.55–3.87 (m, 10H), 3.45–3.30 (m, 6H), 2.94–2.76 (m, 12H), 1.27–1.24 (m, 18H), 1.18–1.12 (m, 12H), 1.07 (s, 9H), 1.02 (s, 9H). ^13^C NMR (100 MHz, CDCl_3_) δ 150.15, 150.00, 149.96, 149.32, 149.20, 147.64, 147.50, 142.44, 142.39, 142.30, 132.52, 132.41, 132.35, 132.26, 128.03, 127.88, 127.86, 127.66, 126.10, 125.86, 125.73, 125.52, 125.39, 125.33, 78.83, 78.60, 66.10, 65.81, 56.32, 55.95, 47.96 (2×CH_2_), 47.94 (2×CH_2_), 34.08, 34.04, 33.96 (2×C), 31.75 (2×CH_2_), 31.73 (2×CH_2_), 31.07 (6×CH_3_), 31.04 (6×CH_3_), 9.76 (2×CH_3_), 9.55 (2×CH_3_). HRMS (ESI+): calculated for C_58_H_87_N_2_O_6_ [M + H]^+^ 907.6564, found 907.6556.

#### 1^5^,3^5^,5^5^,7^5^-tetra-tert-butyl-3^2^,7^2^-bis(3-(dibutylamino)-2-hydroxypropoxy)-1,3,5,7(1,3)-tetrabenzenacyclooctaphane-1^2^,5^2^--diol (5)

Compound **5** was prepared from **2** (39 mg, 0.051 mmol) and di-n-butylamine (1.0 ml), following the procedure used to prepare **3**. The product was obtained as a brown solid (27 mg, 70%). ^1^H NMR (400 MHz, CDCl_3_) δ 8.21 (s, 1H), 7.77–7.66 (m, 1H), 7.09–7.00 (m, 4H), 6.99–6.86 (m, 4H), 4.59–4.52 (m, 2H), 4.50–3.88 (m, 10H), 3.46–3.26 (m, 4H), 3.05–2.82 (m, 4H), 2.80–2.65 (m, 8H), 1.64–1.46 (m, 8H), 1.39–1.30 (m, 8H), 1.29–1.24 (m, 18H), 1.08 (s, 9H), 1.02 (s, 9H), 0.92 (t, *J* = 7.3 Hz, 12H). ^13^C NMR (100 MHz, CDCl_3_) δ 150.23, 149.93, 149.77, 149.48, 149.21, 147.77, 147.47, 142.55, 142.34, 142.17, 133.25, 132.88, 132.80, 132.70, 128.21, 127.87, 127.74, 127.50, 126.42, 125.92, 125.77, 125.69, 125.47, 125.39, 79.76, 79.28, 67.54, 67.06, 57.38, 56.59, 54.38 (2×CH_2_), 54.30 (2×CH_2_), 34.20, 34.12, 33.95, 33.93, 32.51, 32.04, 31.94, 31.74, 31.72 (3×CH_3_), 31.68 (3×CH_3_), 31.18 (3×CH_3_), 31.13 (3×CH_3_), 28.36 (2×CH_2_), 28.17 (2×CH_2_), 20.61 (2×CH_2_), 20.58 (2×CH_2_), 14.08 (2×CH_3_), 14.04 (2×CH_3_). HRMS (ESI+): calculated for C_66_H_103_N_2_O_6_ [M + H]^+^ 1,019.7816, found 1,019.7791.

#### 1^5^,3^5^,5^5^,7^5^-Tetra-tert-butyl-3^2^,7^2^-bis(2-hydroxy-3-(pyrrolidin-1-yl)propoxy)-1,3,5,7(1,3)-tetrabenzenacyclooctaphane-1^2^,5^2^-diol (6)

Compound **6** was prepared from **2** (35 mg, 0.046 mmol) and tetrahydropyrrole (1.0 ml), following the procedure used to prepare **3**. The product was obtained as a white solid (27 mg, 78%). ^1^H NMR (400 MHz, CDCl_3_) δ 7.59 (s, 1H), 7.41–7.35 (m, 1H), 7.08–6.97 (m, 4H), 6.89–6.76 (m, 4H), 5.78 (br, 4H), 4.64–4.51 (m, 2H), 4.28–3.74 (m, 8H), 3.46–3.15 (m, 16H), 2.11–1.99 (m, 8H), 1.29–1.24 (m, 18H), 1.00 (s, 9H), 0.97 (s, 9H). ^13^C NMR (100 MHz, CDCl_3_) δ 150.10, 150.05, 149.96, 149.14, 149.10, 147.67, 147.64, 142.51, 142.36, 132.69, 132.54, 132.51, 132.36, 127.88, 127.79, 127.76, 127.46, 126.16, 125.94, 125.88, 125.73, 125.50, 125.38, 125.26, 78.64, 78.31, 66.68, 66.44, 59.41, 59.11, 54.88 (2×CH_2_), 54.82 (2×CH_2_), 34.11, 34.08, 33.95, 33.94, 31.99, 31.76 (2×CH_2_), 31.72 (6×CH_3_), 31.58, 31.08 (3×CH_3_), 31.06 (3×CH_3_), 23.31 (4×CH_2_). HRMS (ESI+): calculated for C_58_H_83_N_2_O_6_ [M + H]^+^ 903.6251, found 903.6240.

#### 1^5^,3^5^,5^5^,7^5^-Tetra-tert-butyl-3^2^,7^2^-bis(2-hydroxy-3-(methylamino)propoxy)-1,3,5,7(1,3)-tetrabenzenacyclooctaphane-1^2^,5^2^-diol (7)

Compound **7** was prepared from **2** (35 mg, 0.046 mmol) and methylamine (1.0 ml), following the procedure used to prepare **3**. The product was obtained as a brown solid (18 mg, 51%). ^1^H NMR (400 MHz, CDCl_3_) δ 8.64 (s, 1H), 8.08 (s, 1H), 7.08–7.00 (m, 4H), 6.98–6.90 (m, 4H), 5.62 (s, 4H), 4.60 (d, *J* = 19.3 Hz, 2H), 4.30–4.06 (m, 6H), 3.99–3.77 (m, 2H), 3.49–3.20 (m, 8H), 2.75 (d, *J* = 5.5 Hz, 6H), 1.23 (s, 18H), 1.07 (s, 18H). ^13^C NMR (100 MHz, CDCl_3_) δ 149.87, 149.82, 149.68, 148.73, 148.56, 148.11, 147.97, 142.76, 142.55, 133.12, 133.01, 132.85, 132.67, 127.68, 127.60, 127.30, 127.08, 126.39, 126.15, 126.08, 125.91, 125.79, 125.64, 125.51, 78.42, 78.04, 66.75, 66.16, 52.82, 52.51, 34.24, 34.22, 33.95, 33.94, 33.89, 33.78, 32.24, 32.07, 32.02, 31.78, 31.69 (3×CH_3_), 31.67 (3×CH_3_), 31.16 (3×CH_3_), 31.14 (3×CH_3_). HRMS (ESI+): calculated for C_52_H_75_N_2_O_6_ [M + H]^+^ 823.5625, found 823.5591.

#### 1^5^,3^5^,5^5^,7^5^-Tetra-tert-butyl-3^2^,7^2^--bis(3-(ethylamino)-2-hydroxypropoxy)-1,3,5,7(1,3)-tetrabenzenacyclooctaphane-1^2^,5^2^-diol (8)

Compound **8** was prepared from **2** (37 mg, 0.048 mmol) and ethylamine (1.0 ml), following the procedure used to prepare **3**. The product was obtained as a white solid (22 mg, 58%). ^1^H NMR (400 MHz, CDCl_3_) δ 8.64 (s, 1H), 8.53 (s, 1H), 7.07–6.96 (m, 8H), 5.80 (br, 4H), 4.81–4.65 (m, 2H), 4.41–4.10 (m, 6H), 3.95–3.80 (m, 2H), 3.47–3.02 (m, 12H), 1.44–1.34 (m, 6H), 1.26–1.18 (m, 18H), 1.16–1.06 (m, 18H). ^13^C NMR (100 MHz, CDCl_3_) δ 149.99, 149.61, 149.47, 148.73, 148.52, 148.30, 148.07, 142.84, 142.48, 142.38, 133.57, 133.50, 133.21, 133.08, 127.82, 127.14, 127.07, 126.64, 126.18, 125.95, 125.85, 125.74, 125.62, 125.50, 78.23, 77.89, 67.08, 66.87, 50.50, 49.89, 43.60, 43.35, 34.32, 34.29, 33.94, 33.91, 32.65, 32.31, 32.22, 31.99, 31.67, 31.65, 31.63 (3×CH_3_), 31.23 (3×CH_3_), 31.21 (3×CH_3_), 29.78, 11.59, 11.46. HRMS (ESI+): calculated for C_54_H_79_N_2_O_6_ [M + H]^+^ 851.5938, found 851.5928.

#### 1^5^,3^5^,5^5^,7^5^-Tetra-tert-butyl-3^2^,7^2^-bis(2-hydroxy-3-(propylamino)propoxy)-1,3,5,7(1,3)-tetrabenzenacyclooctaphane-1^2^,5^2^-diol (9)

Compound **9** was prepared from **2** (35 mg, 0.046 mmol) and *N*-propylamine (1.0 ml), following the procedure used to prepare **3**. The product was obtained as a white solid (26 mg, 74%). ^1^H NMR (400 MHz, CDCl_3_) δ 8.64 (s, 1H), 8.55 (s, 1H), 7.07–7.03 (m, 2H), 7.03–7.00 (m, 4H), 7.00–6.96 (m, 2H), 4.83–4.64 (m, 2H), 4.41–4.10 (m, 6H), 3.94–3.77 (m, 2H), 3.47–3.09 (m, 8H), 3.03–2.87 (m, 4H), 1.92–1.76 (m, 4H), 1.25–1.20 (m, 18H), 1.16–1.11 (m, 18H), 1.01 (t, *J* = 7.4 Hz, 6H). ^13^C NMR (100 MHz, CDCl_3_) δ 150.04, 149.65, 149.54, 148.77, 148.59, 148.25, 148.04, 142.76, 142.44, 142.34, 133.56, 133.27, 133.14, 127.82, 127.78, 127.16, 127.11, 126.60, 126.18, 125.92, 125.86, 125.72, 125.60, 125.50, 78.23, 77.94, 67.01, 66.89, 50.91, 50.36, 50.27, 50.04, 34.32, 34.29, 33.93, 33.91, 32.66, 32.31, 32.24, 32.01, 31.67, 31.65, 31.64 (3×CH_3_), 31.23 (3×CH_3_), 31.22 (3×CH_3_), 29.78, 19.79, 19.63, 11.42, 11.41. HRMS (ESI+): calculated for C_56_H_83_N_2_O_6_ [M + H]^+^ 879.6251, found 879.6237.

#### 1^5^,3^5^,5^5^,7^5^-Tetra-tert-butyl-3^2^,7^2^-bis(3-(hexylamino)-2-hydroxypropoxy)-1,3,5,7(1,3)-tetrabenzenacyclooctaphane-1^2^,5^2^-diol (10)

Compound **10** was prepared from **2** (47 mg, 0.062 mmol) and n-hexylamine (1.0 ml), following the procedure used to prepare **3** and purified by HPLC to give compound **10** as a brown solid (25 mg, 53%). ^1^H NMR (400 MHz, CDCl_3_) δ 8.63–8.60 (m, 2H), 7.07–6.96 (m, 8H), 4.96–4.66 (m, 3H), 4.40–4.04 (m, 7H), 3.88–3.78 (m, 2H), 3.51–2.84 (m, 14H), 1.91–1.68 (m, 4H), 1.40–1.28 (m, 12H), 1.22 (s, 18H), 1.14 (s, 18H), 0.90–0.86 (m, 6H). ^13^C NMR (100 MHz, CDCl_3_) δ 149.56, 149.47, 148.75, 148.55, 148.29, 148.06, 142.79, 142.44, 133.66, 133.58, 133.31, 133.15, 127.83, 127.10, 127.04, 126.66, 126.19, 126.17, 125.96, 125.83, 125.75, 125.61, 125.49, 100.00, 78.19, 77.84, 67.03, 66.81, 50.84, 50.17, 48.84, 48.47, 34.32, 34.30, 33.93, 33.92, 33.90, 32.71, 32.35, 32.25, 32.01, 31.66, 31.62 (3×CH_3_), 31.38, 31.36, 31.24 (3×CH_3_), 31.22 (3×CH_3_), 31.00, 26.61, 26.58, 26.18, 26.00, 22.54, 22.52, 14.04 (2×CH_3_). HRMS (ESI+): calculated for C_62_H_95_N_2_O_6_ [M + H]^+^ 963.7190, found 963.7176.

#### 1^5^,3^5^,5^5^,7^5^-Tetra-tert-butyl-3^2^,7^2^-bis(2-hydroxy-3-thiomorpholinopropoxy)-1,3,5,7(1,3)-tetrabenzenacyclooctaphane-1^2^,5^2^-diol (11)

Compound **11** was prepared from **2** (44 mg, 0.058 mmol) and thiomorpholine (1.0 ml), following the procedure used to prepare **3**. The product was obtained as a white solid (27 mg, 61%). ^1^H NMR (400 MHz, CDCl_3_) δ 8.76 (s, 1H), 8.33–8.23 (m, 1H), 7.09–6.95 (m, 8H), 4.58–3.89 (m, 10H), 3.49–3.28 (m, 4H), 2.94–2.84 (m, 8H), 2.78–2.60 (m, 12H), 1.26–1.21 (m, 18H), 1.16–1.08 (m, 18H). ^13^C NMR (100 MHz, CDCl_3_) δ 149.93, 149.66, 149.50, 149.17, 148.93, 148.08, 147.82, 142.76, 142.59, 142.53, 133.72, 133.21, 133.20, 128.18, 127.76, 127.59, 127.29, 126.64, 126.12, 125.96, 125.78, 125.65, 125.61, 125.52, 79.63, 78.95, 68.06, 67.67, 61.13, 60.88, 55.78 (2×CH_2_), 55.73 (2×CH_2_), 34.31, 34.25, 33.95, 33.94, 32.88, 32.34, 32.19, 32.02, 31.69, 31.67, 31.64 (6×CH_3_), 31.25 (3×CH_3_), 31.21 (3×CH_3_), 28.19, 28.16. HRMS (ESI+): calculated for C_58_H_84_N_2_O_6_S_2_ [M + 2H]^2+^ 484.2880, found 484.2874.

#### 3^2^,7^2^-Bis(3-(bis(2-hydroxyethyl)amino)-2-hydroxypropoxy)-1^5^,3^5^,5^5^,7^5^-tetra-tert-butyl-1,3,5,7(1,3)-tetrabenzenacyclooctaphane-1^2^,5^2^-diol (12)

Compound **12** was prepared from **2** (49 mg, 0.064 mmol) and diethanolamine (1.0 ml), following the procedure used to prepare **3.** The product was obtained as a white solid (29 mg, 59%). ^1^H NMR (400 MHz, CDCl_3_) δ 8.54–7.85 (m, 2H), 7.09–6.98 (m, 4H), 6.97–6.81 (m, 4H), 4.95 (s, 10H), 4.58 (s, 2H), 4.38–4.16 (m, 4H), 4.04–3.65 (m, 10H), 3.44–3.26 (m, 4H), 3.18–3.04 (m, 4H), 3.00–2.54 (m, 6H), 1.26–1.19 (m, 18H), 1.11–1.00 (m, 18H). ^13^C NMR (100 MHz, CDCl_3_) δ 150.05, 149.58, 149.37, 149.14, 147.85, 147.73, 147.70, 142.72, 133.39, 133.26, 133.09, 132.82, 128.42, 128.13, 127.62, 127.42, 126.69, 126.26, 126.03, 125.85, 125.77, 125.43, 78.89, 78.28, 68.45, 67.74, 59.01 (2×CH_2_), 58.73 (2×CH_2_), 57.99 (2×CH_2_), 57.67 (2×CH_2_), 34.23, 34.21, 33.92, 33.91, 33.89, 32.84, 32.51, 32.20, 31.68 (3×CH_3_), 31.63 (3×CH_3_), 31.20 (3×CH_3_), 31.18 (3×CH_3_). HRMS (ESI+): calculated for C_58_H_88_N_2_O_10_ [M + 2H]^2+^ 486.3214, found 486.3207.

#### 3,3′-((1^5^,3^5^,5^5^,7^5^-Tetra-tert-butyl-3^2^,7^2^-dihydroxy-1,3,5,7(1,3)-tetrabenzenacyclooctaphane-1^2^,5^2^-diyl)bis(oxy))bis(2-hydroxy-*N,N,N*-trimethylpropan-1-aminium) Iodide (13)

A mixture of compound **3** (54 mg, 0.063 mmol) and methyl iodide (1.0 ml) was dissolved in methanol and was stirred overnight at room temperature. After completion of the reaction, the mixture was directly concentrated under reduced pressure. Then the crude product was purified by HPLC to provide compound **13** as a white solid (26 mg, 48%). ^1^H NMR (400 MHz, CD_3_OD) δ 7.28–7.14 (m, 6H), 7.06–7.02 (m, 2H), 4.56–3.90 (m, 12H), 3.85–3.78 (m, 2H), 3.69–3.45 (m, 8H), 3.45–3.34 (m, 14H), 1.29 (s, 12H), 1.22 (s, 6H), 1.19 (s, 6H), 1.06–1.03 (m, 12H). ^13^C NMR (100 MHz, CD_3_OD) δ 150.75, 150.70, 150.66, 150.56, 149.38, 149.34, 144.05, 144.00, 143.98, 134.08, 134.03, 133.97, 129.47, 129.43, 129.34, 129.20, 127.33, 127.16, 127.07, 126.93, 126.55, 126.45, 126.36, 126.34, 79.44, 79.39, 69.62, 69.50, 66.77 (2×CH_3_), 65.25 (2×CH_2_), 55.23 (2×CH_3_), 43.20 (2×CH_3_), 35.01, 35.00, 34.76, 32.46, 32.32, 32.06 (3×CH_3_), 31.54 (3×CH_3_), 31.25, 30.25, 24.40, 24.15, 14.47, 11.44. HRMS (ESI+): calculated for C_56_H_84_I_2_N_2_O_6_ [M − 2I]^2+^ 440.3159, found 440.3152.

#### 2,2′,2″,2‴-((1^5^,3^5^,5^5^,7^5^-Tetra-tert-butyl-1,3,5,7(1,3)-tetrabenzenacyclooctaphane-1^2^,3^2^,5^2^,7^2^-tetrayl)tetrakis(oxy))tetrakis(*N*-(3-(dimethylamino)propyl)acetamide) (16)

Tetraethyl 4-tert-butylcalix[4]arene-O,O′,O″,O‴-tetraacetate (100 mg, 0.10 mmol) was dissolved in tetrahydrofuran, and then lithium hydroxide (48 mg, 2.0 mmol) dissolved in water (2.0 ml) was added. After the mixture was stirred at room temperature for 1.5 h, acetic acid was added to adjust its acidity, then the mixture was diluted with n-butanol and washed twice with water. The organic phase was concentrated under reduced pressure to give crude product **15**, which was directly used for the next step without further purification. Subsequently, **15** was dissolved in DMF (5.0 ml), and then 4-(4,6-dimethoxy-1,3,5-triazin-2-yl)-4-methylmorpholinium chloride (223 mg, 0.81 mmol), DIPEA (0.27 ml, 1.6 mmol) and 3-(dimethylamino)-1-propylamine (203 μl, 0.016 mmol) were added. After stirring overnight at room temperature, the reactant mixture was diluted with n-butanol and washed twice with water. The organic phase was concentrated under reduced pressure, and the crude product was purified by HPLC to yield compound **16** as a brown solid (12 mg, 12%). ^1^H NMR (400 MHz, CD_3_OD) δ 6.92 (s, 8H), 4.85–4.78 (m, 8H), 4.61 (s, 8H), 3.39 (t, *J* = 6.8 Hz, 8H), 2.91–2.77 (m, 8H), 2.72–2.58 (m, 24H), 1.99–1.84 (m, 8H), 1.11 (s, 36H). ^13^C NMR (100 MHz, CD_3_OD) δ 172.09 (4×C), 134.96 (4×C), 134.94 (4×C), 134.88 (4×C), 134.82 (4×C), 127.15 (8×CH), 75.44 (4×CH_2_), 56.71 (4×CH_2_), 43.75 (8×CH_3_), 37.42 (4×CH_2_), 34.98 (4×CH, 4×CH_2_), 31.85 (12×CH_3_), 26.37 (4×CH_2_). HRMS (ESI+): calculated for C_72_H_114_N_8_O_8_ [M + 2H]^2+^ 609.4374, found 609.4359.

### Antibacterial Activity Evaluation

MIC values were determined by the broth microdilution method according to the guidelines established by the Clinical and Laboratory Standards Institute (CLSI). Bacterial cells were prepared on Mueller-Hinton agar (MHA) plates at 37°C for 24 h and adjusted to approximately 1.0 × 10^6^ CFU/ml. Compounds were dissolved in DMSO and water to prepare stock solution, and then diluted to the required concentration in Mueller-Hinton Broth (MHB) medium. The bacterial suspension (100 μl) was added to each well of the 96-well plate and mixed with an equal volume of the two-fold serial dilutions of the compounds (100 μl), then the 96-well plates were incubated at 37°C for 24 h. The MIC values were determined by measuring OD600 and visual inspection. Compared with the negative control group, the MIC value was determined as the lowest sample concentration without bacterial growth. All reported MIC values have been determined repeatedly.

### Hemolytic Activity Evaluation

Fresh rabbit red blood cells (RBCs) were washed three times with PBS, centrifuged at 2,500 rpm for 3 min, and then diluted with PBS to prepare 8% (v/v) suspension. The compounds were dissolved in DMSO (final DMSO concentration of ≤ 0.50%) or PBS, and then diluted with PBS. Two-fold serial dilutions of compounds (100 μl) were incubated for 1 h at 37°C with 100 μl of RBC suspension (∼5.0×10^8^ cells/ml) in a 96-well plate. Then, the mixture was centrifuged at 2,500 rpm for 5 min, 100 μl supernatant was transferred into a 96-well plate and measured the absorbance at 576 nm using BioTek multi-detector microplate reader. RBCs treated with PBS were used as a negative control, and RBCs treated with 2.0% Triton X-100 solution were used as a positive control. The hemolysis percentage was calculated by the following formula: % hemolysis = [(Abs_sample_ − Abs_negative control_)/(Abs_positive control_ − Abs_negative control_)] × 100. All values have been determined at least twice independently with biologically replicates.

### Cytotoxicity Assay

Mouse NCTC clone 929 cells (∼2.0 × 10^4^ cells/well) were incubated in a 96-well plate, and treated with different concentrations of compound **16** at 37°C with 5% CO_2_ for 24 h. Then, 10 μl of CCK-8 reagent was added to each well and incubated at 37°C with 5.0% CO_2_ for 1 h. The absorbance at 450 nm was detected by BioTek multi-detector microplate reader, and the cell viability was calculated by the ratio of OD450 value of compound-treated cells to OD450 value of untreated cells. The experiments were carried out at least twice with biologically replicates.

### SYTOX Green Assay

Bacteria were cultured on MHA plates overnight, then the bacterial cells were washed twice with PBS buffer (10 mM, pH = 7.2) and resuspended in PBS. The absorbance of the suspension at 600 nm was adjusted to 0.20. After mixing with 0.30 μM SYTOX Green, the suspension was cultured in dark. The change of fluorescence intensity was monitored by the BioTek multi-detector microplate reader (excitation wavelength: 504 nm, emission wavelength: 523 nm). After the fluorescence signal was stable, compound **16** dissolved in DMSO was immediately added to the SYTOX Green-treated bacterial suspension, and the change of fluorescence intensity was recorded for about 1 h. All experiments were conducted at least twice with biologically replicates.

### BODIPY™-TR-Cadaverine Displacement Assay

BODIPY™-TR-cadaverine replacement test was used to determine the binding affinity between compound **16** and lipoteichoic acid (LTA). BODIPY™-TR-cadaverine was purchased from ThermoFisher, LTA from *S. aureus* was purchased from Sigma-Aldrich. When the probe binds to LTA, the fluorescence intensity of the probe is quenched. The fluorescence will be significantly enhanced when the probe is replaced and re-dissolved in solution. BODIPY™-TR-cadaverine (final concentration of 5.0 μM) and LTA (final concentration of 10 μg/ml) were mixed in the 24 well plates with Tris buffer (50 mM, pH = 7.4). After 15 min, compound **16** (1×, 2×, 4×, 8×, 16× and 32× MIC) dissolved in DMSO was added, and then the mixture was placed in the dark for 30 min at room temperature. Finally, the fluorescence intensity (excitation wavelength 580 nm, emission wavelength 620 nm) was measured using a Biotek multiple detector microplate reader. These experiments were performed at least twice with biological replications. The percentage of displacement from LTA was obtained according to the following formula: % displacement from LTA = [(F_sample_ − F_0_)/(F_max_ − F_0_)] × 100. F_0_ is the fluorescence intensity of the probe with LTA, and F_max_ is the fluorescence intensity of the probe without LTA and compound **16**.

### Time-Kill Study

Bacterial cells were incubated on MHA plates at 37°C for 24 h, and then adjusted to approximately 1.0 × 10^6^ CFU/ml. The bacterial suspensions were treated with compound **16** at different concentrations (4× and 8× MIC) and were incubated at 37°C. Then, the culture samples (100 μl) were respectively taken from the mixture at 0.5, 1, 2, 4, 8 and 24 h and were serially diluted 10-fold in PBS. The dilutions were plated onto MHA plates and incubated at 37°C for 24 h, then the bacterial colonies were counted. The experiments were repeated at least twice and with biological replicates.

## Data Availability

The original contributions presented in the study are included in the article/supplementary material, further inquiries can be directed to the corresponding authors.
